# Enhancing paramedics procedural skills using a cadaveric model

**DOI:** 10.1186/1472-6920-14-138

**Published:** 2014-07-08

**Authors:** David Lim, Stephen Bartlett, Peter Horrocks, Courtenay Grant-Wakefield, Jodie Kelly, Vivienne Tippett

**Affiliations:** 1 School of Clinical Sciences, Queensland University of Technology, Brisbane, Australia; 2 IHBI Medical Engineering Research Facility, Queensland University of Technology, Brisbane, Australia

## Abstract

**Abstracts:**

**Background**

Paramedic education has evolved in recent times from vocational post-employment to tertiary pre-employment supplemented by clinical placement. Simulation is advocated as a means of transferring learned skills to clinical practice. Sole reliance of simulation learning using mannequin-based models may not be sufficient to prepare students for variance in human anatomy. In 2012, we trialled the use of fresh frozen human cadavers to supplement undergraduate paramedic procedural skill training. The purpose of this study is to evaluate whether cadaveric training is an effective adjunct to mannequin simulation and clinical placement.

**Methods**

A multi-method approach was adopted. The first step involved a Delphi methodology to formulate and validate the evaluation instrument. The instrument comprised of knowledge-based MCQs, Likert for self-evaluation of procedural skills and behaviours, and open answer. The second step involved a pre-post evaluation of the 2013 cadaveric training.

**Results**

One hundred and fourteen students attended the workshop and 96 evaluations were included in the analysis, representing a return rate of 84%. There was statistically significant improved anatomical knowledge after the workshop. Students’ self-rated confidence in performing procedural skills on real patients improved significantly after the workshop: inserting laryngeal mask (MD 0.667), oropharyngeal (MD 0.198) and nasopharyngeal (MD 0.600) airways, performing Bag-Valve-Mask (MD 0.379), double (MD 0.344) and triple (MD 0.326,) airway manoeuvre, doing 12-lead electrocardiography (MD 0.729), using laryngoscope (MD 0.726), using Magill® forceps to remove foreign body (MD 0.632), attempting thoracocentesis (MD 1.240), and putting on a traction splint (MD 0.865). The students commented that the workshop provided context to their theoretical knowledge and that they gained an appreciation of the differences in normal tissue variation. Following engagement in/ completion of the workshop, students were more aware of their own clinical and non-clinical competencies.

**Conclusions**

The paramedic profession has evolved beyond patient transport with minimal intervention to providing comprehensive both emergency and non-emergency medical care. With limited availability of clinical placements for undergraduate paramedic training, there is an increasing demand on universities to provide suitable alternatives. Our findings suggested that cadaveric training using fresh frozen cadavers provides an effective adjunct to simulated learning and clinical placements.

## Background

In Australia over the last decade, there has been a significant paradigm shift in paramedic education from vocational training to university-based tertiary qualification supplemented by practicum [1]. Clinical placements remain an essential opportunity for undergraduate students to practise application of theoretical knowledge and procedural skills in real-world professional practice [2]. However, the paramedic industry in general faces significant mentoring skill and labour shortages that impact on the availability of optimal clinical placement opportunities [3]. As with other health disciplines, simulated learning has been used as a means of providing undergraduate students with a complementary means of skill development [4,5]. However regardless of the fidelity of simulated clinical environments, few practitioners agree that simulation is a satisfactory replacement for in-field practicum [6].

Unlike hospital-based health practitioners, paramedics can find themselves working in challenging, diverse and austere environments which do not lend themselves easily to simulation [7]. Similarly, in emergency situations, they are required to work quickly and efficiently under pressure. Sole reliance on mannequin-based simulation activities and anatomical models is insufficient to prepare a student for the variance of human external and internal anatomy [6].

Paramedic graduates in Australia are expected to be competent in several invasive procedures including maintenance of airway patency through insertion of a laryngeal mask airway, cannulation and needle thoracocentesis to relieve tension pneumothorax [8,9]. To be competent and confident in these procedures, paramedic students require a strong working knowledge of human anatomy and exposure to variations in normal tissue planes and textures as well as minor anatomical variations in internal structures such as the trachea.

The value of cadaveric training has long been recognised as essential in medical education and in allied health training [10-16]. As the scope of paramedic practice expands the gap between the educational requirements of doctors and paramedics for some emergency life sustaining skills arguably becomes less apparent. Holland *et al.* argues that simulated learning is ‘of little or no relevance’ to surgery ‘in which tissue handling and balancing are paramount’ and that ‘cadaveric training provides the opportunity for trainees to understand intimately the anatomy and helps them develop an insight into the technical aspects of the procedures’ [10] (p.110 & 114). Despite this, human cadaveric training has rarely been integrated into tertiary education for paramedics in Australia [17].

The Queensland University of Technology (the "University") Paramedic Science program (the "Program") with the assistance of Medical Engineering Research Facility (MERF), piloted a three-hour fresh frozen human cadaveric training workshop for second-year undergraduate paramedic students in 2012. The workshop aimed to provide the students with an opportunity to refine a range of procedural skills and experience first-hand the differences in human anatomy.

The University’s Bachelor of Paramedic Science is a three-year full-time equivalent on-campus program. The students are provided with three segments of six-week clinical placement opportunities: the first at the end of first year, the second and third placements during the final year of the degree. The focus of the first clinical placement is to expose students to the culture and inherent job requirements of pre-hospital emergency care. The focus of the second and third clinical placements is hands-on application of clinical skills. By the beginning of their second-year in the Program, paramedic undergraduates have been taught procedural skills to maintain airway patency. The students practise such procedural skills on mannequins, namely: ALS Simulator™, MegaCode Kelly™, SimMan Classic™, MegaCode Kid™ and Laerdal Airway Management Trainer™.

For many students, this cadaver-training initiative provided first exposure to a human body and the surface and internal structures critical to their clinical role. In addition, for a significant proportion of students it was their first exposure to the deceased.

We sought to evaluate the cadaveric training program to determine whether or not this addition to the curriculum provided improved acquisition of procedural skills compared to mannequin-based simulation alone. This was achieved by measuring self-perceived competencies pre- and post-workshop. Prior to the cadaveric workshop, students had been exposed to 18 months of traditional simulation-based training and had completed one six-week practicum in the field.

## Methods

The study comprised two sequential steps. The first step involved the conduct of a Delphi study to develop and validate the evaluation instruction. The second step of the project was the pre-post evaluation of the 2013 cadaveric training using the developed evaluation instruction. This study adhered to the RATS guidelines for qualitative research: http://www.biomedcentral.com/authors/rats.

### Ethics

Ethics approval was granted by the Queensland University of Technology Human Research Ethics Committee (UHREC) in June 2013. All participants volunteered to participate and anonymity and confidentiality were assured.

### Donor management

All fresh frozen human cadavers were derived from the MERF donor program and managed by the Facility in keeping with appropriate biological and ethical standards. All cadavers in this program are routinely screened for potentially transmittable infections.

### Participant preparation and support

Paramedic students were prepared for the workshop prior to the event and teaching staff were on-hand to support students as required. Free counselling services were also made available to the students after the workshop if needed. The cadaveric workshops were offered across a teaching-free week and students had the choice to opt-in. Participation in the workshop was not graded.

### Evaluation instrument development

Delphi methodology was utilised to develop an evaluation instrument suitable for our use. The process undertaken was identical to similar work done on assessment of paramedic clinical competence [18]. Delphi method is a flexible iterative research technique useful in developing a descriptive framework for knowledge manipulation activities [19-21].

A Delphi study was conducted with eight members of the teaching staff (the "experts"). These experts were selected because they are involved in teaching across all years of the Paramedic Science curriculum. All staff had expert theoretical and practical knowledge of the clinical competencies required of a paramedic graduates and practicing paramedics. All experts had a minimum of five years’ experience in paramedic tertiary education.

In the first round, a focus group with experts was held to: (a) list the key procedural skills required of a paramedic graduate; and (b) to articulate what the experts wish to achieve from the evaluation. A de-identified Delphi-questionnaire comprised of 26 questions was developed: 10 anatomy knowledge, 3 procedural skills, 9 behaviours, 3 logistical and 1 costs questions.

In the second round, the experts were asked to rate the questionnaire items on a 5-point Likert-scale with a focus on: "How relevant and important are the following to paramedic practice?" The experts were also asked to edit or reword each statement they felt needed revision and provide additional information as appropriate. Consensus was defined as >75% agreement between experts on rating a particular item. After removing overlap responses, the revised questionnaire comprised of 29 questions: 10 anatomy knowledge, 13 procedural skills, 4 behaviours, 1 logistical and 1 directed qualitative questions.

In the final round, a focus group was held with experts to consider the relevance and importance of the statements in regards to paramedic practice and graduate attributes. This yielded the final questionnaire that comprised of 27 questions: 10 anatomy knowledge, 11 procedural skills, 4 behaviours, 1 logistical and 1 qualitative questions.

Two focus groups were held with undergraduate paramedic students who attended the pilot cadaveric training in 2012 to assess the evaluation instrument’s content and face validity [22]. The first face-to-face focus group comprised of three students and the second focus group was conducted with six students online. These focus groups were conducted prior to the 2013 cadaveric workshop. A further one-to-one interview with a 2013 workshop participant was conducted one month after the training to ascertain the validity of the evaluation instrument. The participants were asked to complete the evaluation instrument and discussion was then held to determine whether the items were representative of the constructs understudied and the statements were understandable. Overall, the participants raised no major concerns regarding face or content validity of the instrument.

### Evaluation of 2013 workshop

A pre- and post-test design using the evaluation instrument developed in the preceding step was utilised to assess any changes in students’ anatomy knowledge and confidence in performing procedural skills. The evaluation instrument is contained in Additional file 1.

All 167 second-year Paramedic Science students were eligible to enrol for the workshop. The students were first informed of the cadaveric workshop at the beginning of the semester. The workshop was conducted at MERF. Nine workshops were offered on a teaching-free week in September 2013. Each workshop had a maximum number of twenty students. Each workshop comprised of 10 minutes orientation and instruction on occupational health and safety, a 90-minute anatomical lesson on a formalin-fixed human cadaver, and another 90-minute paramedic procedural skills lesson on a fresh frozen human cadaver.

All students were presented with a pre-evaluation survey form and an information sheet before the workshop. Both pre- and post-evaluation surveys required participants to rate their self-perceived confidence level for eleven procedures. The completed pre- and post-evaluation forms were matched using the last four digits of their student number.

The evaluation instrument comprised questions distributed as follows: ten on anatomy knowledge, eleven on procedural skills, four on behaviours, one on logistical, and one qualitative question (see Table 1 for the anatomy knowledge questions and Table 2 for the procedural skills). For each of the anatomy questions, the students were asked to state whether the statement was "true", "false" or "unsure". For example: *‘the internal thoracic vessels are approximately two finger-widths from the lateral border of the sternum’.* For each of the procedural skills questions, the students were asked to rate on a 5-point Likert scale their level of confidence in performing such procedures on a real patient. Four statements, informed by Ajzen’s theory of planned behaviour [23] asked the students to rate their individual attitudes, perceived behavioural control, and intention on a 5-point Likert-scale. One directed qualitative question enabled students to provide feedback on how the activity could have been improved, and the other enabled students to self-identify the value of human cadaveric training versus high-fidelity mannequin.

**Table 1:** Proportion of students who correctly identified anatomy knowledge based questions before and after the workshop

	**Pre-workshop**	**Post-workshop**	**McNemar test**
The diaphragm and the pericardium are separated by layers of fascia and fluid – false	8 (8%)	62 (65%)	<0.001
The inferior vena cava can be visualised entering the right atrium of the heart – false	13 (14%)	54 (56%)	<0.001
The oesophagus is anterior to the descending aorta in the thorax – false	27 (28%)	26 (27%)	1.000
The right primary bronchus is angled >30 degree laterally – false	23 (24%)	49 (51%)	<0.001
The appendix is approximately 5 cm in length – true	65 (69%)	83 (87%)	0.002
The left/ obtuse margin of the heart consists of both the left atria and left ventricle – false	11 (12%)	61 (64%)	<0.001
The renal veins are larger in diameter than the renal arteries - true	28 (29%)	75 (78%)	<0.001
There are six rectus abdominis muscles – false	53 (55%)	90 (94%)	<0.001
The umbilicus is connected to the liver in an adult – true	4 (4%)	62 (65%)	<0.001
The internal thoracic vessels are approximately two finger-width from the lateral border of the sternum - false	5 (5%)	17 (18%)	0.012

**Table 2:** Students’ self-rated confidence in performing procedural skills on real patient before and after the workshop

	**Pre-workshop**	**Post-workshop**	**MD (SEM)**	**t (p-value)**
Laryngeal mask airway	3.45 (0.972)	4.11 (0.752)	0.667 (0.103)	6.453 (<0.001)
12-lead electrocardiography	3.56 (0.792)	4.29 (0.710)	0.729 (0.072)	10.162 (<0.001)
Laryngoscope	3.41 (0.792)	4.14 (0.752)	0.726 (0.083)	8.796 (<0.001)
Oropharyngeal airway	4.39 (0.701)	4.58 (0.592)	0.198 (0.070)	2.810 (<0.001)
Magill® forceps to remove foreign body obstruction	3.62 (0.936)	4.25 (0.771)	0.632 (0.096)	6.587 (<0.001)
Nasopharyngeal airway	3.91 (0.876)	4.51 (0.682)	0.600 (0.086)	6.937 (<0.001)
Bag-Valve-Mask	4.01 (0.792)	4.39 (0.673)	0.379 (0.084)	4.535 (<0.001)
Double airway manoeuvre	4.18 (0.725)	4.52 (0.598)	0.344 (0.077)	4.482 (<0.001)
Triple airway manoeuvre	4.15 (0.743)	4.47 (0.599)	0.326 (0.081)	4.019 (<0.001)
Thoracocentesis	2.89 (0.993)	4.13 (0.669)	1.240 (0.099)	12.514 (<0.001)
CT-6 traction splint	3.06 (0.982)	3.93 (0.714)	0.865 (0.091)	9.519 (<0.001)

Descriptive statistics were used to summarise the student response data. Cronbach’s α coefficient was used to assess reliability of the evaluation instrument by correlating each item with all other items to show whether each item is related to the one construct [22,24]. The Paired Student’s t-test was used to compare the students’ self-reported confidence levels in performing procedural skills before and after the workshop. McNemar's chi-square was used to ascertain the proportion of correct and incorrect knowledge based questions before and after the workshop. Data analysis was undertaken using *Statistical Package for the Social Science* (SPSS^(R)^, IBM Corp) software, version 21.0 licensed to the University. Content analysis [25] guided the interpretation of qualitative data.

## Results

One hundred and sixty-seven students were eligible to attend and 127 students were enrolled (76%). One hundred and fourteen students presented on their respective days for workshop. There were three missing pre-evaluation and fifteen missing post-evaluation surveys. Consequently, 96 completed pre- and post-evaluation were included in the analysis. The valid completed evaluation represented a return rate of 84%.

### Anatomical knowledge

The ten anatomical items were treated as a single scale. Accordingly, a Cronbach’s α value of 0.30 (pre-) and 0.54 (post-) were calculated. The coefficient alpha for the post-workshop evaluation is considered acceptable for newly developed scales [26].

Eight students (8%) correctly identified the anatomical relations between the diaphragm and pericardium before the workshop, this improved to 62 students (65%) by the end of the workshop (chi-square 2.004, p < 0.001). Similarly, 13 students (14%) before the workshop and 54 students (56%) after the workshop corrected identified the inferior vena cava (chi-square 7.944, p < 0.001). As presented in Table 1, students improved in their anatomical knowledge in regards to bronchi (chi-square 1.153, p < 0.001); thoracic vessels (chi-square 0.019, p = 0.012); obtuse margin of the heart (chi-square 1.754, p < 0.001); appendix (chi-square 3.573, p = 0.002); renal veins (chi-square 0.005, p < 0.001); abdominal muscle (chi-square 1.238, p < 0.001); and umbilicus (chi-square 0.198, p < 0.001). There were however no changes to the students who could correctly differentiate the position of oesophagus to the thorax (pre-workshop: 28%, post-workshop: 27%, chi-square 0.123, p = 1.000).

In the qualitative comments, students reported that the workshop provided context to the theoretical knowledge of anatomical structure. One student wrote, ‘*The anatomy of the cadavers is more realistic in regards to weights* [sic]*, mobility than compared to a mannequin*’.

The anatomy lesson also provided the students with practical context as one student recounted: ‘*Anatomical landmarks. I now understand how all the organs work together*’.

Overall, the students were satisfied with the anatomy lesson: ‘*It is a great experience and I have learnt so much more about practical skills and anatomy then I would have ever learnt on dummies*’.

### Procedural skills

**Figure 1:**
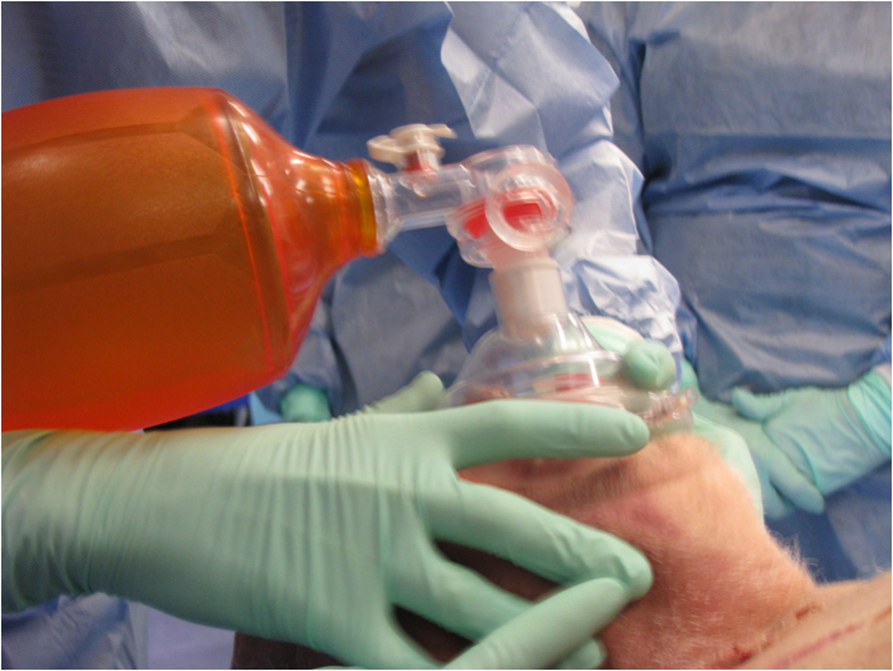
**Students performing Bag-Value-Mask seal on a donor.** Consent for the photographs was obtained from students and representative of the donor.

**Figure 2:**
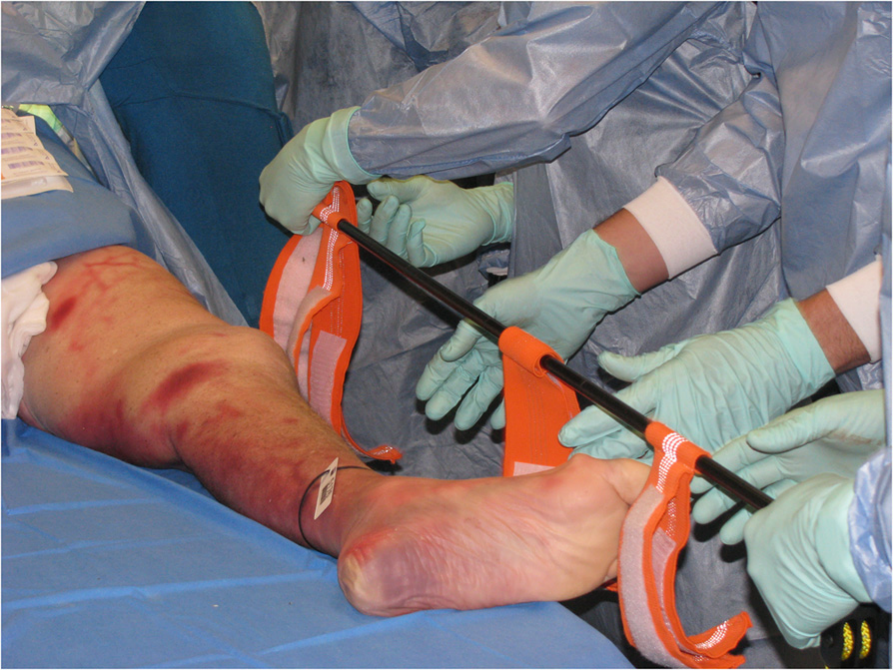
**Students performing CT-6 traction splint.** Consent for the photographs was obtained from students and representative of the donor.

**Figure 3:**
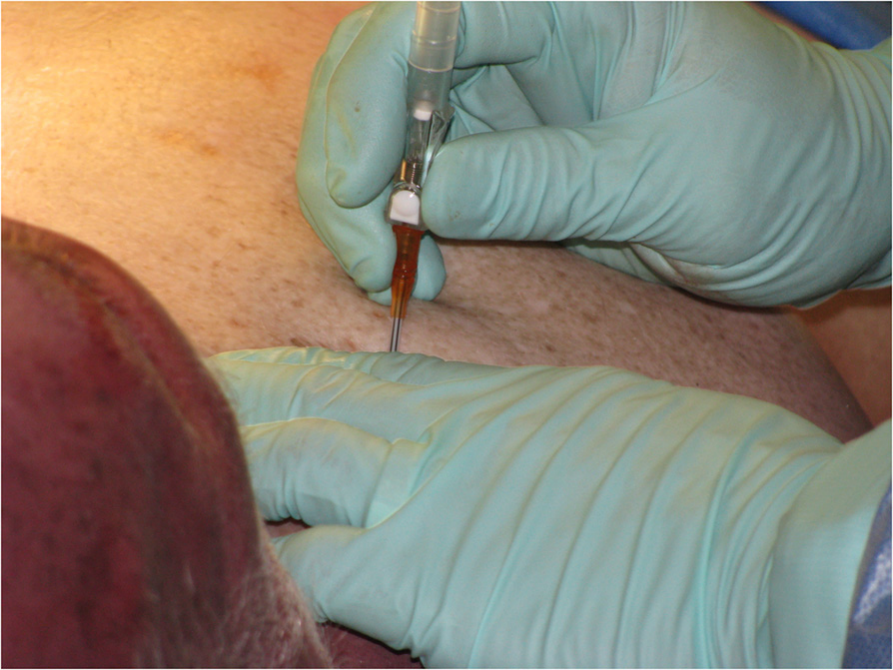
**Students performing thoracocentesis.** Consent for the photographs was obtained from students and representative of the donor.

Students had an opportunity to practise procedural skills on a fresh frozen human cadaver under the supervision and guidance of a tutor qualified as a Queensland Ambulance Service Intensive Care Paramedic. For example, Figure 1 shows students practising how to perform Bag-Value-Mask seal. Figure 2 shows the students applying a CT-6 traction splint and Figure 3 shows the students performing a thoracocentesis.

Students reported a statistical significant improvement in perceived self-confidence after the workshop (see Table 2). Cronbach’s α coefficients for the eleven items were 0.89 (pre-) and 0.91 (post-workshop), indicating very good internal consistency across the items as a single construct [22].

The workshop enabled students to appreciate the differences in normal tissue variation: ‘*it was awesome to put it into perspective and really hone in on the importance of how different and dynamic our bodies are compared to mannequin*’.

At the same time, the procedural skill training made students reflect on their own clinical competencies. As one student wrote, ‘*I learnt that airway manoeuvres are more difficult compared to the mannequin at uni, more practise is needed to integrate my skills*’.

The experience also enabled the students to reflect on their own handling of death and mortality. As one student put it, ‘*Before today I had never seen a deceased person. I think being able to do this in a controlled environment will benefit me when I see someone deceased on road*’.

### Behavioural

Ninety-one percent (91%) of the students Strongly Agreed, and a further 7% Agreed that the workshop had been a powerful learning experience. Likewise, 69% Strongly Agreed and 27% Agreed that the cadaver prompted a realistic response from them. Furthermore, 73% Strongly Agreed and 23% Agreed that as a consequence of this workshop they were more conscious of their own skills and limitations.

Eighty-eight percent (88%) Strongly Agreed and 9% Agreed that they would recommend this workshop to their peers.

This activity was perceived to support preparedness for clinical practice: ‘*I feel better prepared for my first real patient having practised skills on deceased people*’.

## Discussion

Cadaveric training is traditionally regarded to be the gold standard for medical trainees before procedures are performed on patients [10]. Such training is routinely undertaken in medicine and by allied health professions such as physiotherapy, occupational therapy, podiatry, and in some cases nursing [11-16,27]. However, fresh frozen cadaveric training models have not traditionally been integral to paramedic education at either the undergraduate or postgraduate level. This evaluation of undergraduate paramedics’ experience with surface and internal anatomy and procedural skills training using fresh frozen cadavers has demonstrated significant improvement in both anatomical knowledge and self-reported confidence and competence.

Paramedic is an action discipline and students learn in part by doing. The professional competency by which a paramedic is judged is more often on one’s clinical expertise in performing necessary skills. One of the focuses of this evaluation was on the ability to maintain airway patency. This skill is vital for the management of unconscious patients. Failure to effectively manage the airway has obvious and significant negative consequences for the patient. Unmanaged hypoxia can result in cerebral hypoxia, increased intracranial pressure due to uncontrolled hypercapnia and consequently cerebral oedema and secondary brain damage [28]. To successfully perform airway procedural skills, undergraduate paramedics require intimate knowledge of human anatomy and need to be exposed to the normal minor variances in human anatomy. The anatomy of head and neck is complex and almost impossible to simulate realistically on a mannequin [6,10]. Recent advances in human cadaver preservation such as the fresh frozen technique utilised here have enabled the preservation of normal tissue planes, resistance, quality and handling. Fresh frozen cadavers are widely used in surgical teaching [15,27].

In Australia, the move to tertiary qualification has placed an increased reliance on clinical placements as the mechanism for transferring student’s knowledge and skill into novice clinical practice. As is the case in other health disciplines the availability of ambulance clinical placements is under pressure from, increased demands from competing tertiary institutions due to increased student enrolment, and limitation of operational support for clinical placements amongst employers [29].

Simulation learning has been trialled and advocated as adjunct training to augment the demands on clinical placements [4]. However, there are limitations to learning anatomical and procedural skills on low-fidelity mannequins. These include the inability to simulate the treatment of pre-hospital patient in a myriad of different physical and uncontrolled environments; and the inability to realistically replicate the multiplicity of normal variances in human tissues and structural responses [6,10,13]. For instance in the unconscious patient, the ability to adapt airway patency procedure to the position and contraction of the head and neck muscles is critical [30,31]. Similarly, the ability to perform an effective foreign body extraction from the airway requires intimate working knowledge of surface resistance and functional anatomy.

Clinical placement for paramedic students is critical to the education of graduate practitioners who will be expected to make clinical decisions autonomously often in challenging physical circumstances; for example, in confined space. Furthermore, in Queensland, paramedic graduates are able to prescribe and dispense over 25 pharmaceutical agents, and are expected to perform complex and invasive procedures. The current restriction on clinical placement opportunities presents a significant challenge to education providers to ensure that students are able to master the necessary procedural skillsets.

We have demonstrated that our cadaveric learning activity provides an effective tool for the transfer of theoretical knowledge to clinical practice by exposing students to realistic tissue patency, resistance and texture present in fresh frozen human cadavers. The cadaveric training is the adjunct to simulation learning and clinical placement. We believe the cadaveric training also provides reasonable safeguard for patient safety by ensuring that students are as well-prepared for clinical placements in their final year of University and as novice practitioners once they graduate.

Education provider has a duty to prepare students who are job-ready novice practitioners. Other than the best possible opportunity for skill acquisition and refinement, this duty also calls on universities and other education providers to ensure that students are ready for the other inherent aspects of the paramedic profession such as death and dying. Our paramedic student cohort is young and may not have experience with death. Anxiety is a common response to one’s first encounter with a cadaver, this could negatively affect one’s performance [11]. Nonetheless, death and dying is a common aspect of the paramedic’s job. Cadaveric training also provides an opportunity for our students to be exposed to the deceased in a controlled and supportive environment. Students reflected on this experience in the evaluation. This is important in education for paramedic practice and potentially decreases the likelihood of poor patient management as a consequence of fear or anxiety. Paramedics need to react appropriately when confronted with life threatening issues and intervene appropriately with reasonable skill and care. To do this they must be provided with ample opportunities to acquire and cement their skills in the most life-like circumstances possible.

One additional important finding of this study was the fact that students indicated they were more aware of their own limitations as a direct consequence of this workshop. Reflective practice is critical to health care practice as it allows the students to integrate theory with clinical practice and link new to existing knowledge [32]. Reflective practice is a like-long skill which facilitates the transition from student, novice to expert.

## Conclusions

This evaluation demonstrates the value of providing cadaveric training opportunities to undergraduate paramedic students. The model complements simulated training with mannequins and clinical placement. The educational opportunity delivered demonstrated significant improvements in knowledge, interventional skill and attitudes for the participating paramedic students. This was particularly noticeable in regards to appreciation of realistic tissue patency, resistance and texture, and other minor variances in human anatomy.

## Competing interests

The authors declare that they have no competing interests.

## Authors’ contributions

All authors were involved in the development of the evaluation instrument and the drafting of the manuscript. In addition, DL facilitated the ethics and Delphi processes, and did the data analysis; SB, PH and VT contributed to the study concept and critical review of the manuscript; SB, PH and JK provided training and coordination; and CGW recruited for focus groups and did data collection. All authors read and approved the final manuscript.

## Pre-publication history

The pre-publication history for this paper can be accessed here:

http://www.biomedcentral.com/1472-6920/14/138/prepub

## Supplementary Material

Additional file 1 Fresh Frozen Human Cadaveric Training (2013) evaluation. Click here for file
