# Supplementary material for: Enhancing paramedics procedural skills using a cadaveric model
Source: BMC Med Educ. 2014 Jul 8;14:138. doi: 10.1186/1472-6920-14-138 (PMC4099026; doi:10.1186/1472-6920-14-138)
Supplement: Additional file 1 — Fresh Frozen Human Cadaveric Training (2013) evaluation. [file 1472-6920-14-138-S1.docx]

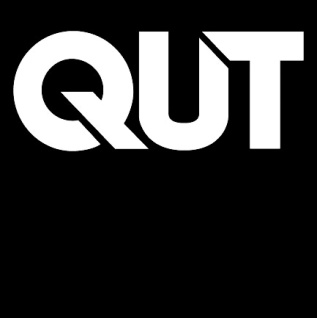
Paramedic Science, School of Clinical Sciences

and

Medical Engineering Research Facility

Fresh Frozen Human Cadaveric Training (2013)

Pre-workshop evaluation

Last four digits of your student number: ___ ___ ___ ___

| 1. Please mark (x) true, false or unsure to the following statements | **True** | **False** | **Unsure** |
| --- | --- | --- | --- |
| 1. The diaphragm and the pericardium are separated by layers of fascia and fluid |  |  |  |
| 1. The inferior vena cava can be visualised entering the right atrium of the heart |  |  |  |
| 1. The oesophagus is anterior to the descending aorta in the thorax |  |  |  |
| 1. The right primary bronchus is angled >30^o^ laterally |  |  |  |
| 1. The appendix is approximately 5cm in length |  |  |  |
| 1. The left/ obtuse margin of the heart consists of both the left atria and left ventricle |  |  |  |
| 1. The renal veins are larger in diameter than the renal arteries |  |  |  |
| 1. There are 6 rectus abdominis muscles |  |  |  |
| 1. The umbilicus is connected to the liver in an adult |  |  |  |
| 1. The internal thoracic vessels are approximately two finger-width from the lateral border of the sternum |  |  |  |

| 1. How confident do you feel in performing the following procedure on a real patient? | **Insecure** | **Unsure** | **Adequate** | **Competent** | **Very Confident** |
| --- | --- | --- | --- | --- | --- |
| 1. Laryngeal mask airway (LMA) |  |  |  |  |  |
| 1. Positioning of 12-lead electrocardiography (ECG) |  |  |  |  |  |
| 1. Use of McGrath laryngoscope |  |  |  |  |  |
| 1. Insert an oropharyngeal (OP) airway |  |  |  |  |  |
| 1. Use McGills forceps to remove foreign body obstruction |  |  |  |  |  |
| 1. Insert a nasopharyngeal (NP) airway |  |  |  |  |  |
| 1. Bag-Valve-Mask ventilation |  |  |  |  |  |
| 1. Double airway manoeuvre |  |  |  |  |  |
| 1. Triple airway manoeuvre |  |  |  |  |  |
| 1. Tension pneumothrax decompression/ thoracocentesis |  |  |  |  |  |
| 1. Apply CT-6 traction splint |  |  |  |  |  |


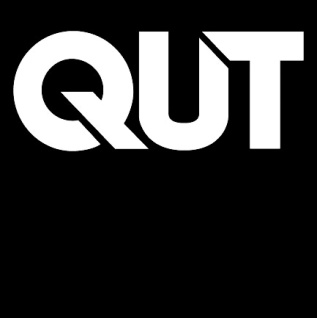
Paramedic Science, School of Clinical Sciences

and

Medical Engineering Research Facility

Fresh Frozen Human Cadaveric Training (2013)

*Post-workshop evaluation*

Last four digits of your student number: ___ ___ ___ ___

| 1. Please mark (x) true, false or unsure to the following statements | **True** | **False** | **Unsure** |
| --- | --- | --- | --- |
| 1. The diaphragm and the pericardium are separated by layers of fascia and fluid |  |  |  |
| 1. The inferior vena cava can be visualised entering the right atrium of the heart |  |  |  |
| 1. The oesophagus is anterior to the descending aorta in the thorax |  |  |  |
| 1. The right primary bronchus is angled >30^o^ laterally |  |  |  |
| 1. The appendix is approximately 5cm in length |  |  |  |
| 1. The left/ obtuse margin of the heart consists of both the left atria and left ventricle |  |  |  |
| 1. The renal veins are larger in diameter than the renal arteries |  |  |  |
| 1. There are 6 rectus abdominis muscles |  |  |  |
| 1. The umbilicus is connected to the liver in an adult |  |  |  |
| 1. The internal thoracic vessels are approximately two finger-width from the lateral border of the sternum |  |  |  |

| 1. How confident do you feel **now** in performing the following procedure on a real patient? | **Insecure** | **Unsure** | **Adequate** | **Competent** | **Very Confident** |
| --- | --- | --- | --- | --- | --- |
| 1. Laryngeal mask airway (LMA) |  |  |  |  |  |
| 1. Positioning of 12-lead electrocardiography (ECG) |  |  |  |  |  |
| 1. Use of McGrath laryngoscope |  |  |  |  |  |
| 1. Insert an oropharyngeal (OP) airway |  |  |  |  |  |
| 1. Use McGills forceps to remove foreign body obstruction |  |  |  |  |  |
| 1. Insert a nasopharyngeal (NP) airway |  |  |  |  |  |
| 1. Bag-Valve-Mask ventilation |  |  |  |  |  |
| 1. Double airway manoeuvre |  |  |  |  |  |
| 1. Triple airway manoeuvre |  |  |  |  |  |
| 1. Tension pneumothrax decompression/ thoracocentesis |  |  |  |  |  |
| 1. Apply CT-6 traction splint |  |  |  |  |  |

**Cont/...**

| 1. In your opinion, what are the values of fresh frozen cadaveric training versus high fidelity mannequin for undergraduate paramedic training? {clinically/ personally/ humanistically} |
| --- |
|  |

| 1. Please mark (x) your level of agreement | **Strongly agree** | **Agree** | **Unsure** | **Disagree** | **Strongly disagree** |
| --- | --- | --- | --- | --- | --- |
| 1. I learned a great deal from this activity |  |  |  |  |  |
| Can you be specific? Further comment? | | | | | |
| 1. The cadaver prompted realistic responses from me |  |  |  |  |  |
| Can you be specific? Further comment? | | | | | |
| 1. I am more conscious of my own skills and limitations as a result of this activity |  |  |  |  |  |
| Can you be specific? Further comment? | | | | | |
| 1. I am likely to recommend this course to my peers |  |  |  |  |  |

| 1. The activity could have been improved by: |
| --- |
|  |

| 1. Any other comment? |
| --- |
|  |

Thank you ☺

*Your insight helps us to improve this workshop*
